# Parallelized, real-time, metabolic-rate measurements from individual *Drosophila*

**DOI:** 10.1038/s41598-018-32744-0

**Published:** 2018-09-27

**Authors:** Anthony Fiorino, Dakotah Thompson, Swathi Yadlapalli, Chang Jiang, Orie. T. Shafer, Pramod Reddy, Edgar Meyhofer

**Affiliations:** 1 Department of Mechanical Engineering, University of Michigan, Ann Arbor, MI 48109 USA; 2 Department of Molecular, Cellular and Developmental Biology, University of Michigan, Ann Arbor, MI 48109 USA; 3 Department of Biomedical Engineering, University of Michigan, Ann Arbor, MI 48109 USA

## Abstract

Significant recent evidence suggests that metabolism is intricately linked to the regulation and dysfunction of complex cellular and physiological responses ranging from altered metabolic programs in cancers and aging to circadian rhythms and molecular clocks. While the metabolic pathways and their fundamental control mechanisms are well established, the precise cellular mechanisms underpinning, for example, enzymatic pathway control, substrate preferences or metabolic rates, remain far less certain. Comprehensive, continuous metabolic studies on model organisms, such as the fruit fly *Drosophila melanogaster*, may provide a critical tool for deciphering these complex physiological responses. Here, we describe the development of a high-resolution calorimeter, which combines sensitive thermometry with optical imaging to concurrently perform measurements of the metabolic rate of ten individual flies, in real-time, with ~100 nW resolution. Using this calorimeter we have measured the mass-specific metabolic rates of flies of different genotypes, ages, and flies fed with different diets. This powerful new approach enables systematic studies of the metabolic regulation related to cellular and physiological function and disease mechanisms.

## Introduction

Metabolism is defined as the sum of all physical and biochemical processes in living organisms that either produce or consume energy^1,2^. As part of their regular metabolic activity, cells and organisms must constantly replenish their energy supply through complex metabolic pathways such as the uptake and breakdown of nutrients, cellular respiration *etc*. resulting in a diverse set of reaction intermediates and products (metabolites). Since energy input is essential, metabolic processes are involved in virtually all cellular processes and the inevitable heat losses provide an integrative signature of cellular and organismal activity^1,2^. Early work on metabolic measurements in *Drosophila* centered around the rate of living theory^3^, with various studies reporting evidence to support^4,5^ or refute^6,7^ this hypothesis. More recently, significant evidence has been accumulating to suggest that various common human diseases such as cancer^8,9^, obesity^10^ and diabetes^11^, and aging^12,13^ involve abnormal metabolic states. Therefore, obtaining insights into the regulation of metabolism, from cells and individual model organisms, is crucial to not only understand the overall functioning of cells and cellular mechanisms but also for developing new approaches to treating metabolism-linked diseases^14^.

A number of techniques, including quantification of metabolites, respirometry and direct calorimetric measurements, have been utilized to characterize the metabolic output and state of biological systems ranging from collections of cells to whole organisms. The first strategy, broadly speaking, is based on profiling (i.e. quantifying the concentrations of) specific, low molecular weight metabolites that, as fundamental constituents of the key biochemical pathways, serve as metabolic indicators^15,16^. But this strategy has significant limitations: (1) Destructive sample preparation prevents continuous, time-resolved measurements and (2) relating the detected biomarkers to biological mechanisms remains challenging and uncertain^15^. The second major approach, respirometry, quantifies the metabolic rate of biological systems from measured O_2_-consumption or CO_2_-production rates^17,18^. Using a flow-through experimental configuration and sensitive CO_2_-gas analysis, time-resolved measurements from single, small model organisms like *Drosophila* are feasible^19,20^, but this approach may suffer from potential errors in metabolic estimates^21^ when respiratory coefficients are uncertain or switching between aerobic and anaerobic metabolic pathways occurs, and is challenging to parallelize. In the final approach, direct calorimetry, the aerobic and anaerobic metabolic activity is determined from the heat production of the system^17,22–25^, which quantifies the total metabolism activity because all cellular processes (e.g. energy conversion, gene expression, motility) have finite efficiencies resulting in characteristic heat dissipation. However, current direct calorimetric methods do not have the desired sensitivity, response time, throughput or physiological compatibility to conduct metabolic studies from individual, small model organisms.

Here, we present a calorimetric method with high resolution and fast response time that enables quantification of the metabolic activity of individual fruit flies (*Drosophila melanogaster*). Our calorimeter setup is capable of simultaneous measurements of metabolic heat output and activity levels from ten individual fruit flies in real-time. In this study, we choose to employ *Drosophila melanogaster* as it has emerged as an important model system in metabolic research^26–28^ due to the metabolic pathways shared with mammals^27^ and the relative ease with which the fly’s genome can be manipulated. Using the calorimeter, we measured the basal and the average metabolic heat output of flies of different genotypes, ages and flies fed with different diets. Our studies demonstrate that our sensitive calorimeter can precisely quantify the metabolic activity of individual *Drosophila* and will enable further studies investigating metabolic disorders associated with many pathologies including aging, circadian disruption, mitochondrial dysfunction among others.

## Results

### Calorimeter design and characterization

**Figure 1:**
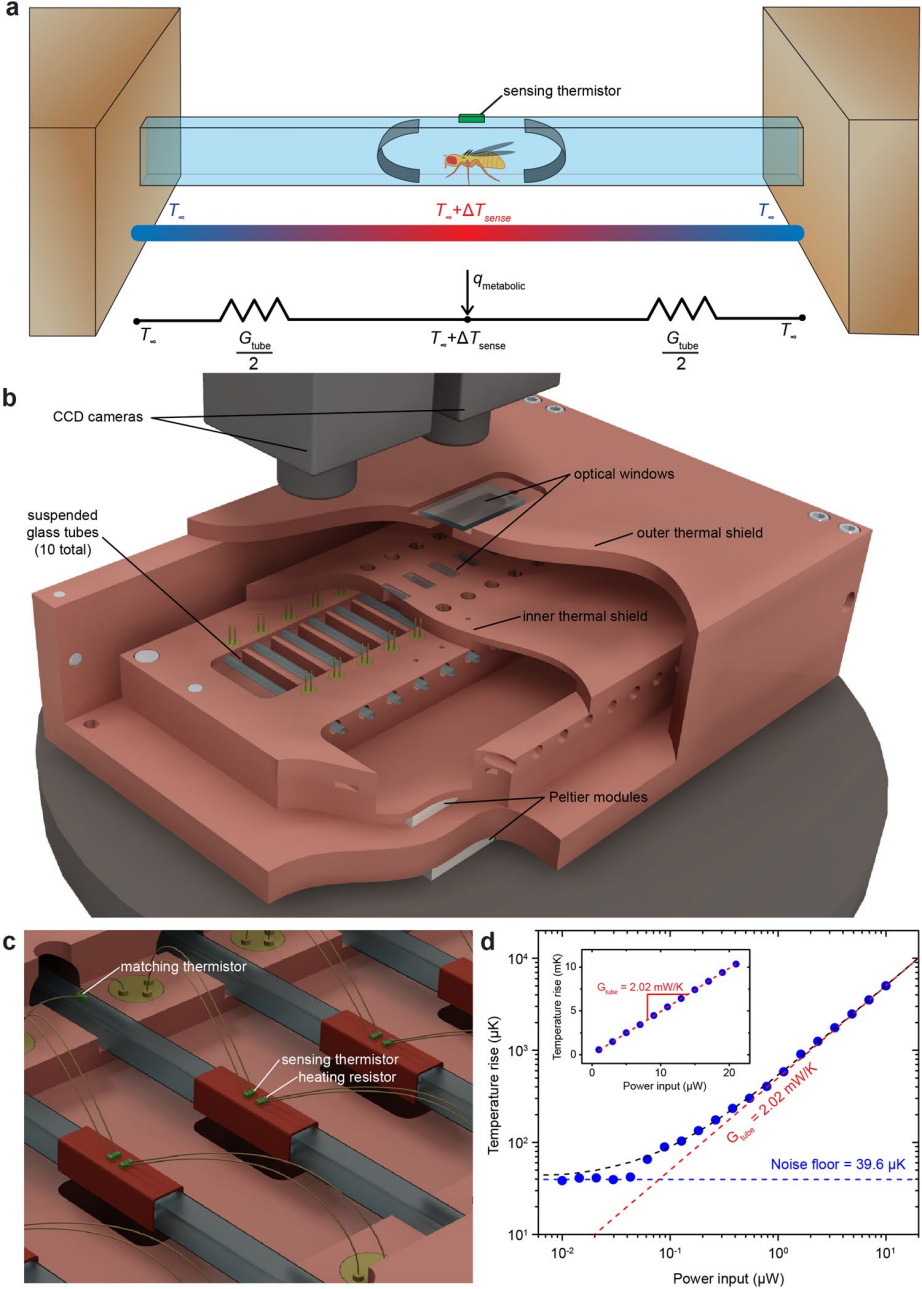
Calorimetric platform. (**a**) Schematic illustration of the calorimeter’s working principle. The fly is contained at the center of a glass tube (2 × 2 mm^2^ inner cross section). The high-sensitivity thermistor detects the small temperature increase, Δ*T*_sense_, due to the fly’s heat output, *q*_metabolic_. The thermal conductance of the glass tube, *G*_tube_, is the primary pathway for heat transfer as shown in the thermal resistance network. (**b**) Rendering of the entire system. Two independently-controlled sets of Peltier modules maintain the temperature of the inner and outer thermal shields. Two CCD cameras optically image the ten measurement chambers. All ten flies breathe air from a temperature-controlled, humidified air reservoir (Methods). (**c**) Detailed view of selected calorimeter tubes with a sensing thermistor for measuring Δ*T*_sense_ and a heating thermistor for calibrating the conductance (*G*_tube_). (Methods). Copper tape ensures isothermal conditions at the center of the tube. (**d**) Plot of the temperature rise (Δ*T*_sense_) vs. controlled electrical heat input (*q*_Joule_) for a representative calorimeter showing that heat flows as small as 100 nW can be reliably detected. Inset: Same as (**d**) but on linear axes. The inverse of the slope yields *G*_tube_ = 2.02 mW/K.

Measurement of the heat output enables direct quantification of metabolic activity of organisms as all cellular processes are accompanied by characteristic heat dissipation. In our study, we developed a calorimetric platform that is capable of measuring the heat output from an individual fly. Our calorimetric platform consists of two major components: First, the calorimetric sub-system which constitutes of ten suspended glass tubes (VitroCom S102), each of which is instrumented with a sensitive thermistor and serves as an experimental chamber for a single fly, and second, an optical imaging system to track the movement of each fly in real-time (Fig. 1). Each calorimetric chamber features a 30 mm long glass tube suspended in air and supported at both ends by a temperature-controlled copper shield (Fig. 1a). The basic working principle of the calorimeters is as follows: For metabolic measurements, a single fly is loaded into and confined to the central segment of a glass tube (Fig. 1a). The heat output associated with the fly’s metabolic processes causes the temperature at the center of the tube to increase by Δ*T*_sense_, which is detected by the high-resolution thermistor (Fig. 1a,c). The heat output, *q*_metabolic_, can be directly obtained from *q*_metabolic_ = *G*_tube_ × Δ*T*_sense_, where *G*_tube_ is the thermal conductance of the glass tube. The thermal conductance *G*_tube_ was determined for each individual tube in a separate calibration measurement (see Methods) and was found to be 2.01 mW K^−1^ for the representative tube shown in Fig. 1d. With *G*_tube_ calibrated for each tube, the key to resolving small heat outputs is the ability to detect small temperature changes, which, in this system, is limited by temperature drift of the thermal shields and 1/*f* electronic noise^29–31^. To attenuate temperature drift in our system, we employ active PID-feedback control of both the inner and outer shields (Fig. 1b) and use an ac-driven bridge circuit (see Methods) to minimize the effects of 1/*f* electronic noise. To further suppress the effects of temperature drift, we integrate a second thermistor, referred to as the “matching thermistor” (Fig. 1c), at the base of the glass tube to account for fluctuations in the calorimeter’s temperature due to external perturbations (see Methods). These techniques make possible excellent temperature resolution (±30 μK) (Methods, Supplementary Fig. S6b), which translates into a calorimetric resolution of ~100 nW (Fig. 1d). Finally, the temporal resolution of the calorimeter is set by the thermal time constant of the calorimeter device and is ~50 s.

**Figure 2:**
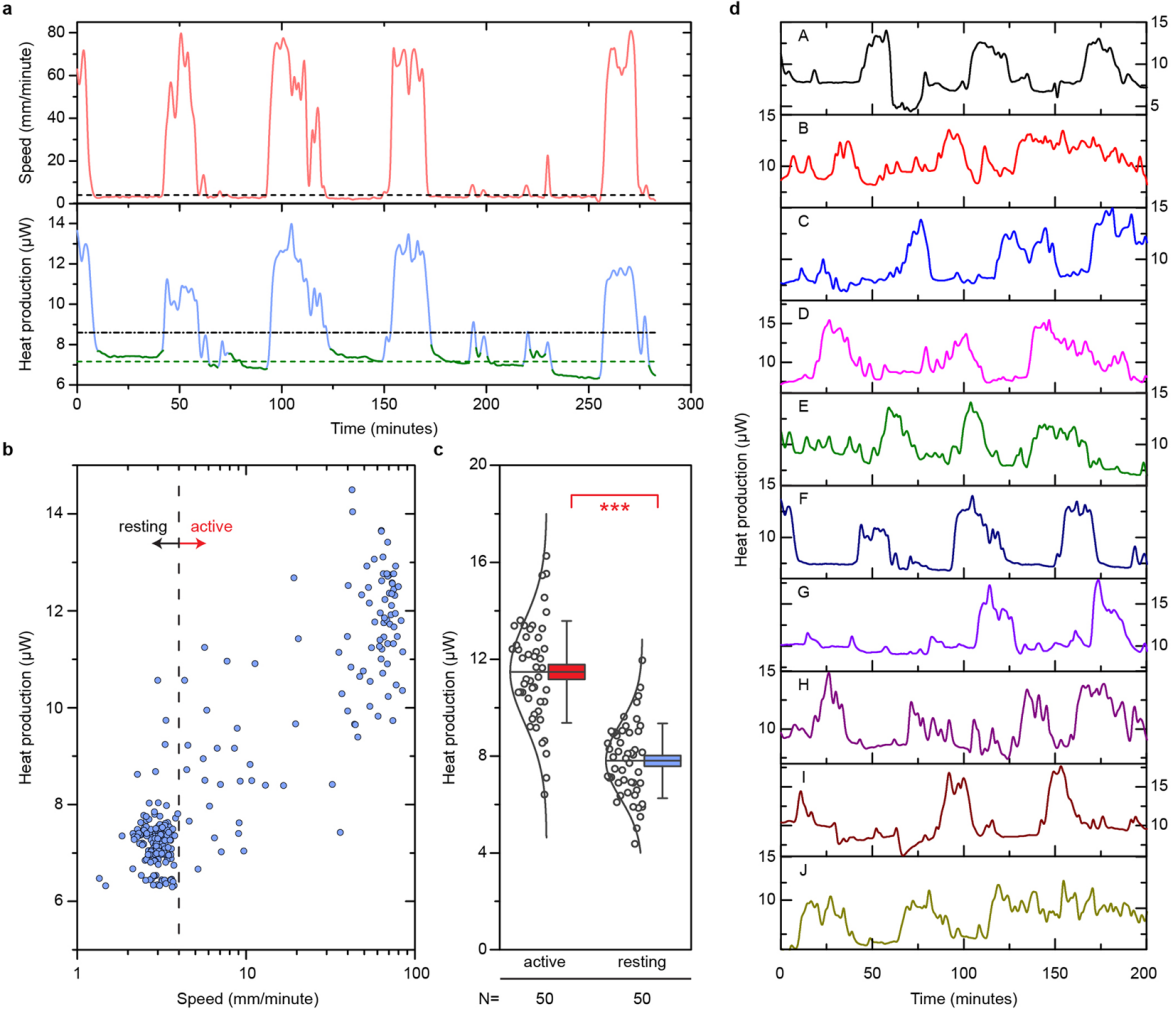
Metabolic measurements. (**a**) Time traces showing the speed of locomotion (upper panel) and heat production (lower panel) of a single *Canton S* (female, 3 days after eclosion) over the course of an experiment. The black dashed line in the upper panel delineates the 4 mm/min threshold for defining the rest state, and the green portions of the heat production trace (lower panel) indicate the times for which the rest condition is met. The dashed green line indicates the average basal heat production while the black dashed-dotted line represents the total average heat production for this fly. (**b**) Heat production plotted against activity level for the same single *Canton S* fly from (a). The dashed vertical line indicates the threshold for the rest condition. (**c**) Heat production data for a population of 50 individual *Canton S* flies averaged during periods of high (>50 mm/min) and low (<4 mm/min) activity. The horizontal line represents the mean for a sample. Box boundaries indicate the standard error of the mean, and error bars represent the standard deviation of the sample. The open circles to the left of the boxes signify the average heat production for the individual flies. ***p < 0.001. (**d**) Heat production data for ten flies (A–J) during a single experiment. Fly F is shown in (**a,b**).

To correlate measured metabolic output of flies to their activity level, we used the calorimeter’s imaging system to record the movement of all flies in real time (Methods). Two CCD cameras (Fig. 1b), positioned above the setup, continuously record the flies and track, via a custom-developed algorithm (see Methods), their position and activity level (quantified as distance traversed per time). We ensured that the calorimeter is thermally unperturbed during imaging by employing very low illumination levels (632 nm wavelength, power density of ~0.5 µW/cm^2^) and optical windows (Fig. 1b) that block infrared radiation (most radiative heat transfer at room temperature occurs in the infrared range^32^). The upper panel of Fig. 2a shows a representative activity trace from a single *Canton S* fly (a commonly used wildtype strain of *D. melanogaster*) while the lower panel presents the simultaneously obtained metabolic output. These observations suggest that (1) the fly’s activity level and metabolism are, as expected, highly correlated and (2) the fly is predominantly either resting or highly active, with almost no intermediate levels of activity.

**Figure 3:**
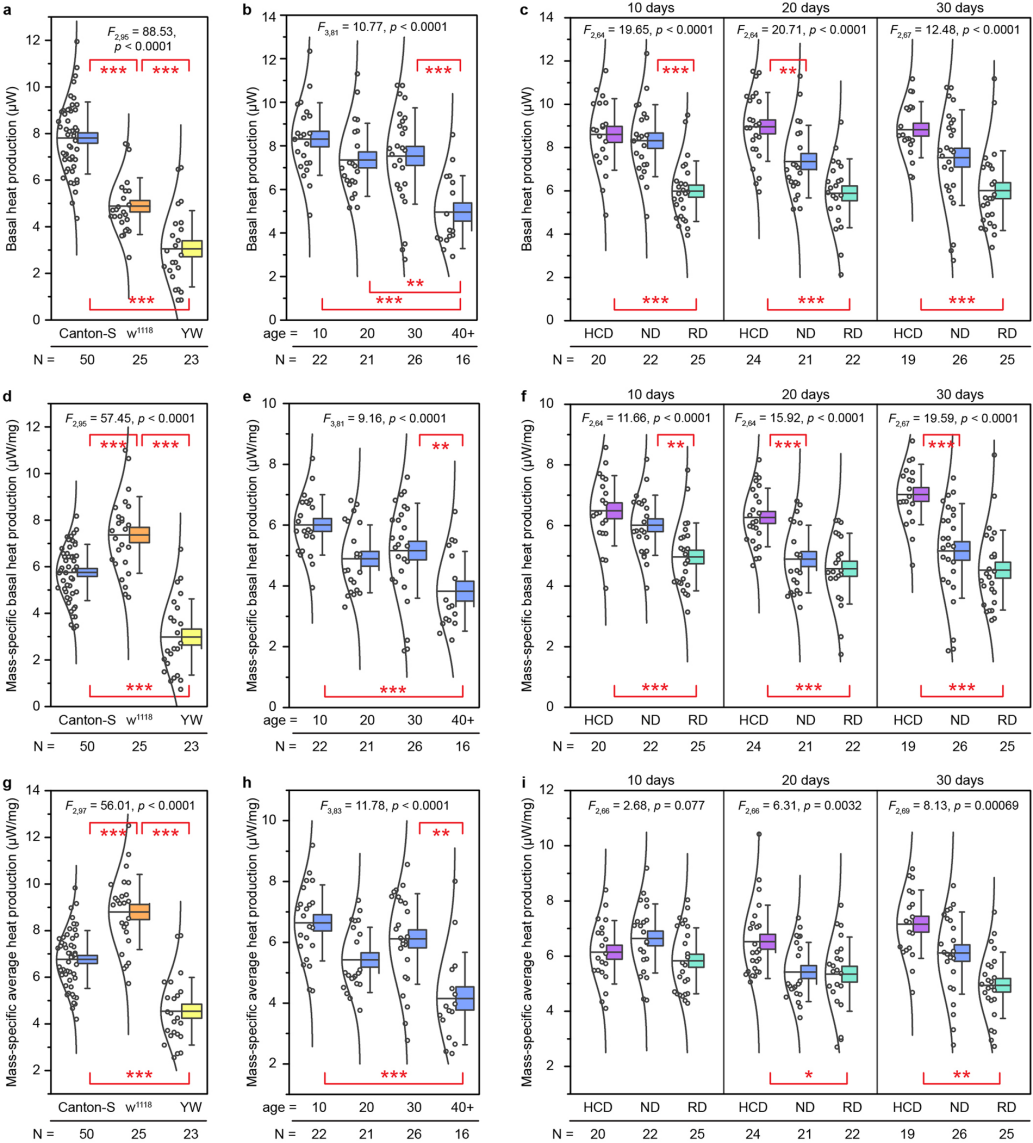
Basal and average metabolic heat production rates. **(a–c)** Basal heat production as measured for: **(a)** the three *Drosophila* genotypes considered: *Canton S*, *w*^1118^, and *yw*, **(b)** *Canton S* flies of different ages, and **(c)** *Canton S* flies on high calorie (HCD), normal (ND) and restricted diets (RD) at different ages. **(d–f)** Mass-specific basal metabolic rate of flies (see Supplementary Fig. S2) of different: **(d)** genotypes, **(e)** ages, and **(f)** dietary conditions. **(g–i)** Mass-specific average heat production for the same flies as in **(a–c)**. Shown are the mean (horizontal line), standard error (box) and standard deviation (error bars). The open circles to the left of the boxes represent the average heat production for individual flies and *N* indicates the sample size. ANOVA results are indicated on each panel; Tukey’s tests are indicated pairwise: ^*^p < 0.01, ^**^p < 0.005, ^***^p < 0.001.

In order to better visualize the relationship between metabolism and activity level, we present in Fig. 2b a plot of the heat output data as a function of fly activity for the same *Canton S* fly from Fig. 2a. As expected, the heat output increases for higher levels of activity, and two clusters of points can be identified: one tight cluster at low activity and metabolism (resting) and another at high activity and metabolism (active). This observation holds true for other genotypes as well (Supplementary Fig. S1). For the analysis of the basal metabolic rate, we define a fly to be at rest if its center of mass moves <4 mm over a 1-minute time period, and in Fig. 2c we compare the metabolic data during rest and high activity (movement >50 mm/min) levels for a sample of 50 *Canton S* flies. We find that metabolic output increases by ~50% when the fly is active (Fig. 2c). To ensure that this behavior is innate to the fly and not governed by the fly’s response to changes in the environment or an artifact of the measurement technique, we show the metabolic rate of 10 flies recorded concurrently in a single experiment in Fig. 2d. The fact that the data from all 10 calorimeters do not track each other indicates that the calorimetric signal from each tube is independent and not influenced by an external stimulus. Thus, we conclude that simultaneous, time-resolved calorimetric and optical measurements enable simultaneous recording of heat output and identification of time periods when the flies are at rest, allowing us to quantify the basal metabolic rates of individual flies. Moreover, the same setup can be used to measure the average metabolic activity of flies, which is an average of all the heat outputs of individual flies through the course of the experiment, irrespective of whether the flies are resting or moving (Fig. 3).

### Effects of genotype, age, and diet on metabolic rate

To establish our system’s ability to precisely measure metabolic heat output of individual *Drosophila*, we performed measurements on three standard laboratory wild-type strains of *Drosophila*: *Canton S*, *w*^1118^, and *yellow white* (*yw*). In these experiments, we used 3-day old mated female flies raised on sugar yeast food (normal diet (ND), Supplementary Table S1) and measured the basal metabolic output (Fig. 3a) of the flies, as described earlier. We also normalized each fly’s basal heat production by its body mass^33^ (Supplementary Fig. S2) to determine the mass-specific heat production (Fig. 3d). The averaged mass-specific basal metabolic heat production of *Canton S* flies is 5.78 ± 0.17 µW/mg), and that of *w*^1118^ flies and *yw* flies is 7.36 ± 0.24 µW/mg and 2.99 ± 0.34 µW/mg, respectively (Fig. 3d). The metabolic rates of individual flies within a genotype fall within a narrow range and individual data points are shown in the figure panels. Interestingly, the range of metabolic rates within a genotype cannot be explained by differences in body mass as indicated by the lack of a strong positive correlation between metabolic rate and body mass (Supplementary Fig. S7). This lack of a correlation between metabolic rate and body mass has also been observed in a previous study, which compared the metabolic rates of 52 recombinant inbred lines^34^. Based on these observations, we expressed metabolic rates per whole animal (Fig. 3a) as well as per mg (Fig. 3d). Moreover, we also find that the genotype with the smallest average mass (*w*^1118^, Supplementary Fig. S2) does not exhibit the smallest basal metabolism (Fig. 3a) as one may expect from simple isometric or allometric scaling^35,36^ (Supplementary Fig. S3).

Our data suggest that there is a difference in the basal heat production per whole animal (Fig. 3a) and mass-specific basal heat production (Fig. 3d) of flies belonging to different genotypes. Furthermore, we also measured the mass-specific average metabolic heat output (Fig. 3g), which is an average of the heat outputs of the fly during the course of the experiment irrespective of the fly’s activity level. The mass-specific average metabolic measurements also showed significant differences between flies of different genotypes. To our knowledge, our calorimeter setup is the first system, which can simultaneously record both the metabolic heat output and locomotor activity of individual flies, while the majority of previous studies measured average metabolic heat output of a collection of flies with no activity monitoring. Nevertheless, our results are consistent with previous studies which reported a metabolic heat production of ~7.2 µW/fly^34,37^. Our results differ somewhat from another previous study, which reported mass-specific metabolic rates of 17.3 ± 0.3 µW/mg that were measured from a collection of flies with no activity monitoring^38^. These differences may be due to the genotypes of the various flies, as well as differences in experimental conditions with regard to calorimeter resolution and activity monitoring used in the studies.

To evaluate if our calorimeter and experimental procedures are suitable for studying the relationship of metabolism and aging, we performed experiments on flies of different ages. Specifically, we compared the basal and average metabolic rates of *Canton S* flies 10, 20, 30, and 40 days after eclosion. Our results indicate that absolute (Fig. 3b), mass-specific basal (Fig. 3e), and mass-specific average (Fig. 3h) metabolic rates vary with the age of the flies. Specifically, both the basal and average mass-specific heat production for flies 40 days and older is significantly lower compared to 10-day old or 30-day old flies (Fig. 3e,h). Such a decline might be expected as mitochondrial damage and a decrease in mitochondrial activity has been shown to play a critical role in normal aging and correlates with the development of a wide range of age-related diseases^39–41^. In addition, previous studies on *D. subobscura* have also revealed that key respiratory enzymes significantly decreased with age^42^. However, previous studies in *D. melanogaster* failed to demonstrate any significant metabolic decline from middle (15 day old) to old age (45 day old), although the same study reported a significant drop in metabolic rate between day 5 and later ages^34^. The reasons for these differences remain unclear, but the cellular and physiological mechanisms that limit the longevity of recombinant inbred *D. melanogaster* adult flies^42^ are likely distinct from those affecting the age-dependent metabolism in wild-type flies. Also, mitochondrially-driven, age-dependent metabolic declines might only be apparent at higher activity levels. From the data presented here we conclude that our instrument is well-suited to study age-dependent metabolic factors in *D. melanogaster*.

 Finally, we used our calorimeter to examine the effects of dietary restriction on metabolism. Dietary restriction (DR), the limitation of food intake below the normal level without malnutrition, has been shown to extend life span in various model organisms, ranging from the unicellular yeast^43^ to nematodes^22,44^, fruit flies^38^, rodents^45^, and primates^46,47^. However, how DR affects the organismal metabolic rate is not well understood, with some studies showing an increase^43,48^ in metabolic output while others show no change^38^. In order to illustrate the suitability of our approach for addressing aspects of this question, we performed basal and average metabolic rate measurements on adult *Canton S* flies of several different ages, raised on food of varying caloric quality. Our data for 10 day-old flies indicate that a fly’s diet affects both its absolute (Fig. 3c) and mass-specific basal metabolic rate (Fig. 3f). Specifically, the flies on high-calorie diets (HCD, Supplementary Table S1) have higher basal metabolic rates (Fig. 3c) and mass-specific basal metabolic rates (Fig. 3f) than DR flies. This observation is found to also be true for 20 and 30 day-old flies (Fig. 3c,f). Interestingly, when we compared the mass-specific average metabolic rates of flies fed with different diets, we only detected significant differences between flies fed high calorie diet (HCD) and restricted diet (RD) at later ages (Fig. 3i). These results differ from previous reports, which either had coarser resolution or indirectly probed metabolism, that suggested that DR has no effect on mass-specific metabolic rate^38^ or even increases it^43,48^. A possible explanation for these different results is the experimental conditions used to assay metabolic rates: in the previous study groups of flies were confined to small chambers, and it is not clear whether the flies were limited in their ability for normal movement (see Discussion for details). The mechanisms of how metabolism is affected by diet are not well known, and it will therefore be interesting to investigate the links between metabolism, diet and longevity in future studies.

## Discussion

In this study, we developed a sensitive calorimeter with integrated imaging capabilities that allows one to simultaneously record the metabolic measurements and activity levels of individual fruit flies in real-time. Our instrument enables precise measurements of resting as well as average metabolic rates of ten individual flies in parallel. We have characterized the calorimeter device and performed first proof-of-principle experiments to validate full functionality of the instrument for metabolic measurements on individual flies.

Our experimental setup consists of a calorimetric sub-system, an imaging sub-system, and a temperature control sub-system. The calorimetric sub-system consists of a tube for holding *Drosophila*, a sensing thermistor that is capable of detecting small temperature increases in response to the heat output of the fly, and circuitry to measure temperature changes detected by the sensing thermistor. The imaging sub-system incorporates a low intensity LED illumination and CCD cameras to record the locomotor activity of the flies. The temperature control sub-system comprises a heat shield, a temperature controller, and a Peltier device. Using this setup, we can measure the metabolic output of multiple, individual flies with high resolution (~100 nW), which broadly matches or exceeds the resolution of continuous indirect, respirometry-based calorimeters that quantify the production of CO_2_ with a sensitive gas analyzer^17^. A detailed comparison of the calorimetric resolution of our system versus respiratory-based systems is difficult as many parameters, including chamber volume, mixing, flow rates, the presence of water vapor, gas analyzer and multiplexing, impact the measurements and functional properties of the instruments. Clearly, both approaches offer sufficient resolution to record metabolic data for individual flies. Our system certainly excels at continuously and simultaneously recording the activity levels of numerous flies as a function of time, enabling us to measure the basal metabolic output of flies at rest, as well as average metabolic output. An additional strength of the current setup is that the metabolic rate can be measured under both resting conditions as well as under different activity levels.

We characterized the metabolism of three commonly used wild-type strains of *D. melanogaster* and reported metabolic rates in the same range as previously published reports^34,37^. Furthermore, our data showed, as expected,  that activity and metabolic rates are highly correlated for all the genotypes we tested. We further investigated how the metabolic rates of animals is affected under conditions which result in increased longevity, for instance dietary restriction. Dietary restriction is the only physiological treatment reported to extend lifespans in a wide variety of animals^49^. The mechanisms by which dietary restriction causes life span extension are not well understood, but there is enormous interest in the field of aging to study the links between metabolism and extended life span. The “rate of living hypothesis” was first put forth by Pearl about a century ago linking metabolic output of an organism to its longevity^3^. The current understanding in the field is that the free radicals produced during aerobic respiration cause cumulative oxidative damage that eventually result in aging^50^, and the conditions that would tend to decrease an organism’s metabolic rate (low temperature, dietary restriction) tend to increase longevity. To date the strongest evidence of temperature effects on longevity comes from studies demonstrating that ectotherms reared at lower temperature live longer than warm-temperature animals^51^. This effect of temperature on longevity is thought to be mediated through the effects of temperature on metabolic rate, with metabolic rates typically changing two- to three-fold for every 10 K change in temperature^52^. However, the causal mechanisms linking metabolic rate and longevity are not well understood and numerous, often conflicting, results have been published on the relationship between these two variables.

In our current studies, we found that dietary restricted flies tend to have lower mass-specific metabolic rates compared to flies fed high-calorie diet at later ages. These results are different than previously published results, which might be explained by the differences in the fly genotypes used and the resolution and experimental conditions of the calorimeter. Interestingly, there are no differences in average metabolic rate between dietary restricted flies and flies fed a normal diet at earlier time points. We also note that we have conducted all our experiments at the same time of the day as accumulating evidence has shown that the clock transcriptionally controls a remarkable fraction of the genome^53,54^, suggesting a powerful link between circadian clocks and metabolism. A majority of the previous studies have not taken this point into consideration and have not controlled for possible time-of-day effects on metabolism of organisms, which could also account for some of the discrepancies between the data in our study and the published literature.

In summary, we report a novel tool capable of direct measurements of the metabolic rate in *Drosophila*. Our approach, which supports concurrent measurements on multiple individual fruit flies for improved throughput, enables systematic studies of the effects of diet and gene expression on metabolism and will greatly facilitate new studies seeking to clarify the link between metabolism and lifespan/aging. Further, our method can also be adapted to perform long-term studies to understand the links between circadian clocks and metabolism^55,56^ by integrating food and environmental stimuli into the apparatus.

## Methods

### Thermometry

In order to measure small temperature changes, Δ*T*_sense_ in the sensing thermistor embedded into the calorimeter (Fig. 1a), we custom-built an ac-driven Wheatstone bridge circuit for differential resistive thermometry (Supplementary Fig. S5). Broadly, the principle is to measure, with high-resolution, changes in the sensing thermistor resistance *R*_T_ relative to the reference (matching) thermistor resistance *R*_M_. To accomplish this, we excite the Wheatstone bridge with a 200 mV rms voltage at 19.9 Hz using a waveform generator (Agilent 33210 A). High-stability (±2 ppm/K) 100 kΩ fixed resistors (Vishay Foil Resistors S Series) are used in the upper half of the bridge so that the bridge voltage *V*_B_ = *V*_M_ − *V*_A_ (Supplementary Fig. S5) is insensitive to temperature changes of the circuit itself. Stable (±20 ppm/K) potentiometers (TT Electronics/BI Model 7280 Series) with a resistance of 5.1 kΩ, are connected in series with the fixed resistors so that the amplitudes of *V*_M_ and *V*_A_ can in principle be balanced to within a few nV at the operating temperature (296 K). Further, the phase difference between *V*_M_ and *V*_A_ are also matched using shunt capacitors *C*_M_ and *C*_A_ (Supplementary Fig. S5). We achieve this by inserting stable (C0G/NP0, ±30 ppm/K) fixed capacitors (TDK FK Series) into sockets connected in parallel with the thermistors. By iteratively tuning the potentiometer resistance and shunt capacitance, we can reliably minimize the difference in instantaneous voltages to be <20 nV. The difference operation *V*_M_ − *V*_A_ is performed using an instrumentation amplifier (Analog Devices AD524) that amplifies *V*_B_ (gain = 100) before it is filtered using a custom-built 6-pole low pass Butterworth filter. Nine additional half-bridge legs are connected in parallel and referenced to the matching thermistor leg (Supplementary Fig. S5) so that all ten signals can be recorded independently using one circuit. The amplified and filtered signals are recorded using LabView and post-processed using a custom-developed, Fast Fourier Transform-based lock-in technique scripted in MATLAB. The temperature resolution of the circuit was quantified to be ± 17 μK (Supplementary Fig. S6c) and does not set the noise floor of the measurement, which is limited by temperature drift.

### Feedback control of temperature

Temperature control of the calorimetric system (Fig. 1b) is achieved by employing a feedback loop that measures and controls the temperature of two thermal shields, called the outer and inner thermal shields. The outer thermal shield (Fig. 1b) is a hollow 24.8 × 16.5 × 7.6 cm^3^ box made of 1.3 cm-thick copper. To maintain the outer shield temperature to within ~1 mK of the 296 K set point, we employ a commercial PID temperature controller (Laird Technologies TC-XX-PR-59), which receives feedback from a thermistor (US Sensor Corp. USP12838) which is permanently bonded into the copper wall using epoxy (3 M Scotch-Weld Epoxy Adhesive 2216 B/A). The controller can either heat or cool the outer shield by driving current through four Peltier modules (Laird Technologies 56590-502) connected in series. The opposite side of the Peltier modules are in good thermal contact with a large steel plate that serves as a heat sink for the outer shield control loop.

The inner thermal shield (Fig. 1b) is a 17.1 × 12.7 × 2.5 cm^3^ two-piece copper clamshell structure that encloses the calorimeter and air reservoir cavities when it is assembled. Controlling the temperature of the inner shield requires high resolution temperature measurement from the feedback thermistor, so a custom control scheme is used. A commercial thermistor (US Sensor Corp. USP12838) is permanently bonded into the inner shield using epoxy (3M Scotch-Weld Epoxy Adhesive 2216 B/A). This thermistor is incorporated along with three fixed resistors into a Wheatstone bridge circuit (Supplemental Fig. S4). The circuit is supplied with a 1 V rms voltage at 37.5 Hz (Hewlett Packard 33120 A) to avoid coupling to the calorimeter circuit. The amplitudes and phases of *V*_L_ and *V*_R_ are balanced when the thermistor is at 296 K by tuning series potentiometers (TT Electronics/BI Model 7280 Series) and shunt capacitors *C*_L_ and *C*_R_ (TDK FK Series), in the same manner described for the calorimeter circuit. An instrumentation amplifier (Analog Devices AD524) performs the difference operation and amplifies the ac signal (gain = 100), which is deconvolved using a lock-in amplifier (Stanford Research Systems Model SR830). We implemented a MATLAB-scripted PID controller, which reads the bridge voltage from the lock-in and drives a current through four Peltier modules (Laird Technologies 56590-502) connected in series using a commercial current source (Keithley Instruments 6221). This scheme is capable of maintaining a stable inner shield temperature to within ±100 µK (Supplementary Fig. S6a).

### Humidity

One major factor that contributes error to calorimetric measurements is evaporative cooling caused by water loss through the specimen’s exoskeleton, respiration, and/or the excretion of feces/urine^8^. In fact, we observe that under certain conditions the evaporative heat loss can overwhelm the calorimeter signal, leading to an apparent net cooling. To minimize evaporative heat loss, we humidify the test chamber to nominally 100% relative humidity by including a layer of deionized water in the air reservoir (Fig. 1b). In addition to suppressing the evaporative heat loss, the elevated humidity has the added benefit of extending the timespan over which flies can survive in the calorimeter by reducing their water loss. An undesirable consequence of the humidification is that it extends the time it takes for the system to stabilize after flies are loaded for an experiment. To expedite the humidification, we raise the temperature of the inner thermal shield to 299 K for 10 minutes after flies are loaded before returning the inner thermal shield to the 296 K set point. Steady state operation is reached within ~2 hours.

### Optical system

Optical access through the thermal shields is necessary to determine periods during which the flies are at rest, so that the basal heat production can be extracted. However, opening holes in the thermal shields also permits undesirable thermal coupling between the ambient environment and the calorimeter via radiation exchange. To reduce thermal drift in the heat production signal, we use glass bandpass filters (Thorlabs, Inc. FGS900S) as optical windows. These filters transmit in the wavelength range 315–710 nm but absorb longer-wavelength infrared radiation (which primarily contributes to thermal exchange) before it reaches the sensitive thermometry in the calorimeter. The windows were diced and epoxied (3 M Scotch-Weld Epoxy Adhesive 2216 B/A) into narrow viewports to limit the view factor from the calorimeter to the environment.

To perform our imaging, we use low intensity 632 nm LED illumination. Two CCD cameras are positioned above the outer thermal shield to collect images of the illuminated flies during the experiment. Each camera is responsible for collecting light from five calorimeter tubes simultaneously, and the images are logged at two frames per second using LabView software. The resulting images are post-processed using a custom-developed algorithm that estimates the fly center of mass for each individual frame so that activity level can be quantified.

### Thermal conductance measurement

The thermal conductance of the calorimeter characterizes the degree to which the measurement chamber is thermally isolated from the surrounding environment, and a correct estimate of the thermal conductance is imperative to accurately quantify the metabolic heat rate of the fly. To perform this characterization, the center suspended region of an empty calorimeter is first heated by Joule heating a 5 kΩ heating resistor (Fig. 1d) glued to the tube outer wall. A dc voltage is applied across this resistor using the output channel of a National Instruments DAQ connector block (BNC-2090A), and the heat dissipation is given by *q*_diss_ = *V*^2^/*R*, where *V* is the supplied voltage and *R* is the thermistor resistance. The resulting temperature rise of the center suspended region is then measured using the sensing thermistor (Fig. 1a,c). The measured temperature rise of the calorimeter for various heat inputs is plotted in Fig. 1d, revealing that the temperature rise scales linearly with the heat input for *q*_diss_ > 100 nW. The inverse slope of the best-fit line through the data points in Fig. 1d inset characterizes the thermal conductance of the calorimeter, which in this case is found to be 2.02 mW/K.

### Data analysis

Basal metabolic heat production was calculated for an individual fly by averaging its heat production during periods of rest (Fig. 2a). One-way ANOVA was performed on the collected data to compare the effects of genotype, age, and diet on metabolic rate. If the ANOVA indicated a significant difference in samples at the *p* < 0.01 level, then a Tukey’s honest significant difference test was used to identify which means differed significantly (Fig. 3).

### Fly husbandry and mass measurement

Flies were raised on cornmeal-yeast-sucrose food (recipe 4 from Bloomington Drosophila Stock Center’s website http://flystocks.bio.indiana.edu/Fly_Work/media-recipes/caltechfood.htm) under a 12:12 light:dark cycle at 25 °C and 60–70% humidity. To study the effect of different diets on metabolic rate, we transferred 3-day old, mated female flies (used for all studies) into vials containing restricted or high-calorie diet (recipes provided in Supplementary Table 1). The mass of each fly was determined right before the start of the experiment by anesthetizing the fly by briefly exposing it to ice and placing it on a digital scale. The experiments were conducted around the same time every day to control for the time-of-day variations. The flies used in the study, *Canton S*, *w*^1118^, *yw*, were obtained from the Bloomington Drosophila Stock Center.

## Electronic supplementary material

Supplementary Information
