# Supplementary material for: Parallelized, real-time, metabolic-rate measurements from individual Drosophila
Source: Sci Rep. 2018 Sep 27;8:14452. doi: 10.1038/s41598-018-32744-0 (PMC6160429; doi:10.1038/s41598-018-32744-0)
Supplement: Supplementary file 1 — Supplementary Information [file 41598_2018_32744_MOESM1_ESM.pdf]

Supplementary information

Parallelized, real-time, metabolic-rate measurements from individual *Drosophila*

Anthony Fiorino[*,1], Dakotah Thompson*[*,1], Swathi Yadlapalli[2], Chang Jiang[1], Orie. T. Shafer[2], Pramod Reddy[+,1] and Edgar Meyhofer[+,1,3]*

[1]Department of Mechanical Engineering, University of Michigan, Ann Arbor, MI 48109, USA

[2]Department of Molecular, Cellular and Developmental Biology, University of Michigan, Ann Arbor, MI 48109, USA

[3]Department of Biomedical Engineering, University of Michigan, Ann Arbor, MI 48109, USA

[*]These authors contributed equally to this paper.

[+]email: pramodr@umich.edu, meyhofer@umich.edu

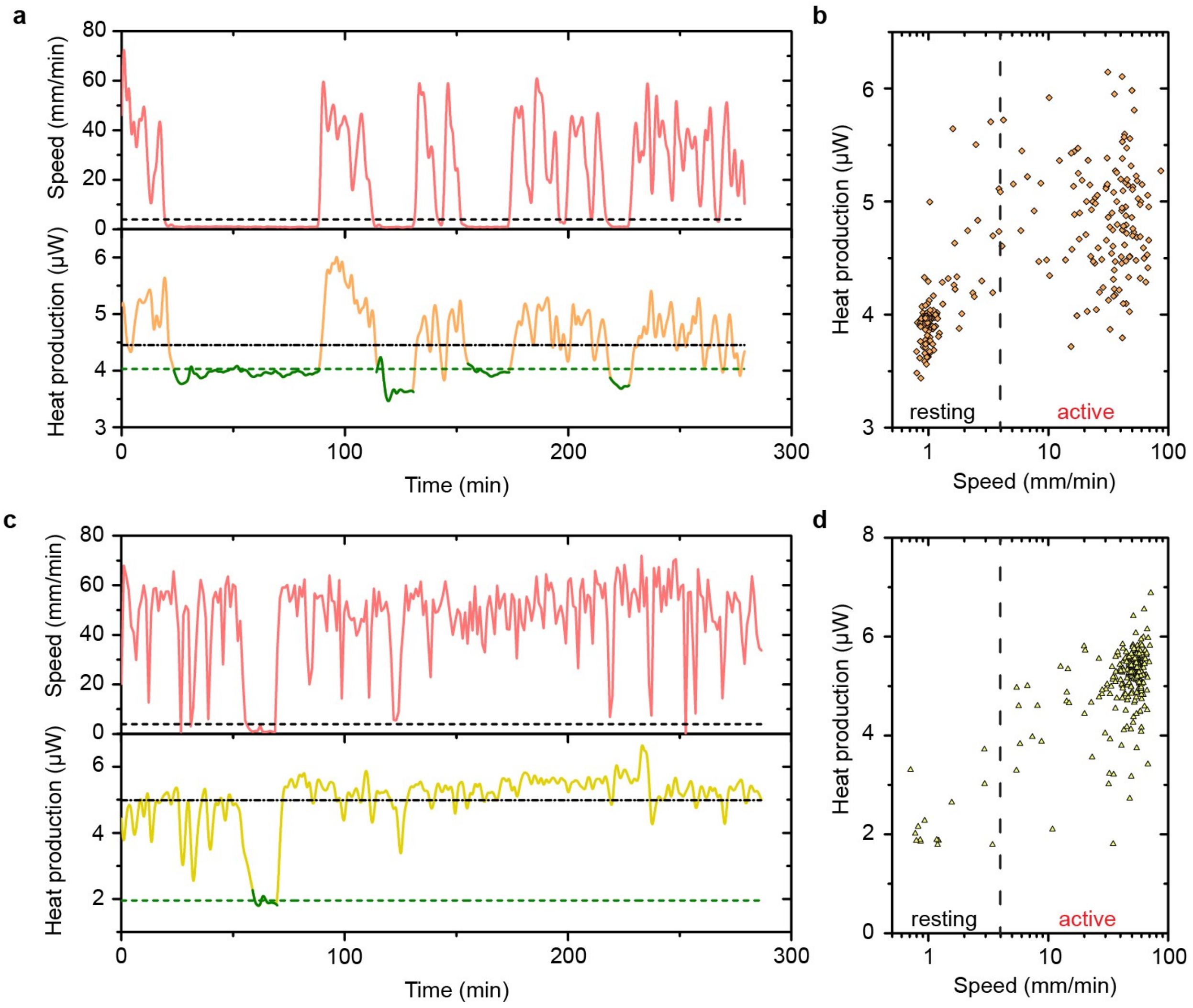

Supplementary Fig. S1 | (**a**) Time traces showing a single w[1118] (mated female, 3 days after eclosion) fly's activity level (upper panel) and absolute heat production (lower panel) over the course of an experiment. The black dashed line in the upper panel delineates the 4 mm/min threshold of the rest state. The green lines in the lower panel indicate heat production during rest; this data is averaged to determine the basal heat production rate (dashed green line). The black dashed-dotted line represents the total average heat production for this fly. (**b**) Heat production plotted against activity level for the same single w[1118] fly from (a). The dashed vertical line indicates the threshold for the rest condition. (**c**) Same as in (a), but for a YW (female, 3 days after eclosion) fly. (**d**) Same as in (b), but for the single YW fly from (c).

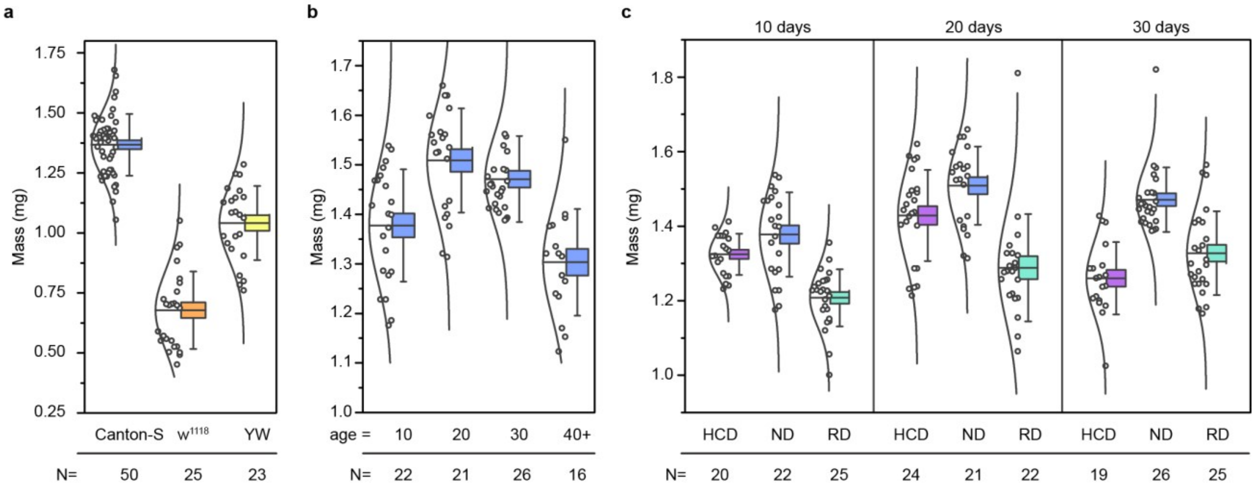

Supplementary Fig. S2 | (**a**) Fly mass measured for the flies in the three *Drosophila* genotype samples (Canton-S, w[1118], and YW). All flies were female. The mean, standard error of the mean, and standard deviation for each genotype are indicated by a horizontal line, box, and error bars, respectively. N represents the sample size. (**b**) Same as in (a), but for female Canton-S flies 10, 20, 30 or 40+ days past eclosion. (**c**) Same as in (a) and (b), but for female Canton-S flies entrained on high-calorie (HCD), normal (ND) or restricted (RD) diets. Surprisingly, flies fed on a HCD weigh less than those entrained on ND.

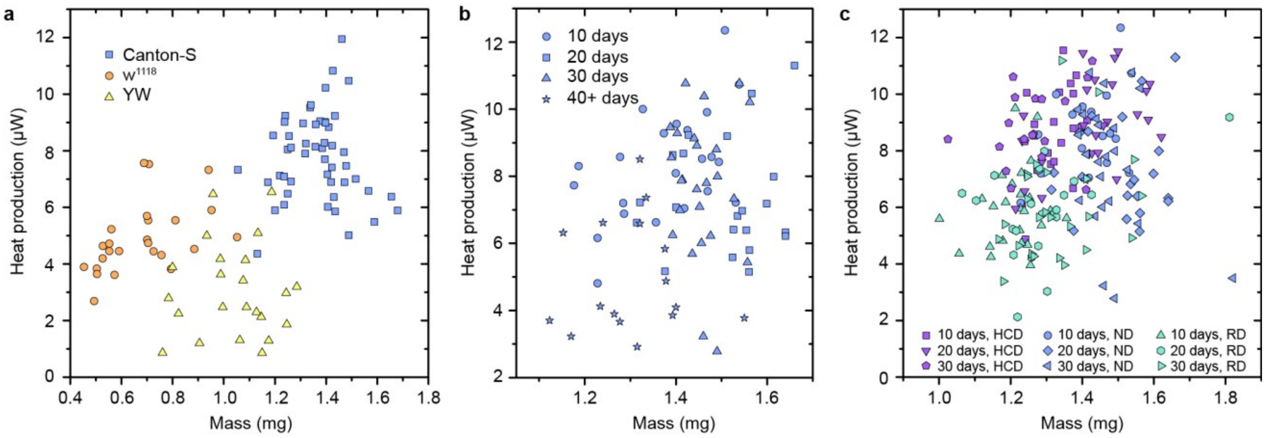

Supplementary Fig. S3 | (**a**) Average basal heat production plotted against fly mass for Canton-S, w[1118], and yellow-white (YW) flies from **Fig. 3a,b** and **Supplementary Fig. S2a**. (**b**) The same as in (a), but for the Canton-S flies from **Fig. 3c, d** and **Supplementary Fig. S2b**. (**c**) The same as in (a) and (b), but for the Canton-S flies entrained on different diets from **Fig. 3e, f** and **Supplementary Fig. S2c**. It can be seen that the heat production for the flies in our samples does not exhibit straightforward allometric scaling.

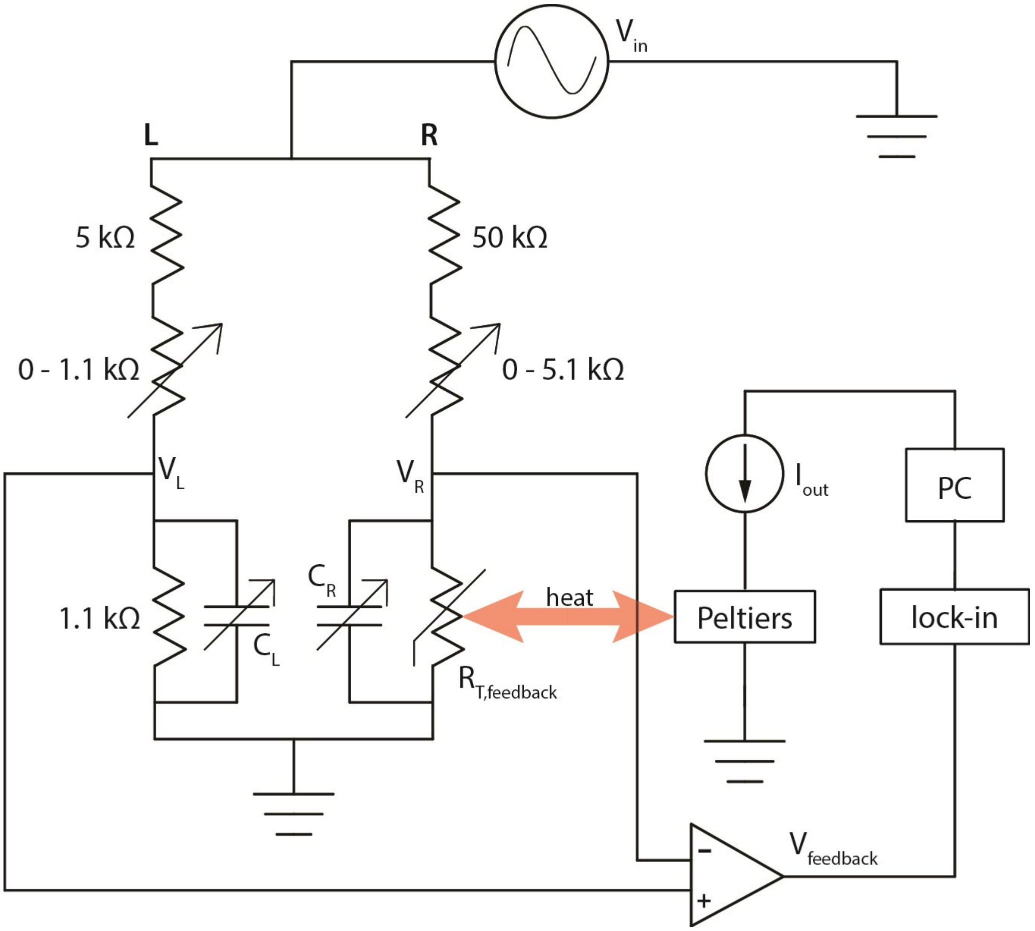

Supplementary Fig. S4 | Circuit diagram and control schematic for the temperature control scheme of the inner thermal shield. When the resistance of the thermistor $R_{\mathrm{T,feedback}}$ changes due to a change in the shield temperature, an ac voltage difference V_L-V_R develops across the bridge. A lock-in amplifier is used to deconvolve the output signal $V_{\mathrm{feedback}} = 100 \times (V_L\text{-}V_R)$ from the operational amplifier and report it to a PC software-based PID controller via a GPIB interface. The software controls the current I_{out} through a set of Peltier coolers in order to rebalance the circuit. See also Online Methods and **Supplementary Fig. S6a**.

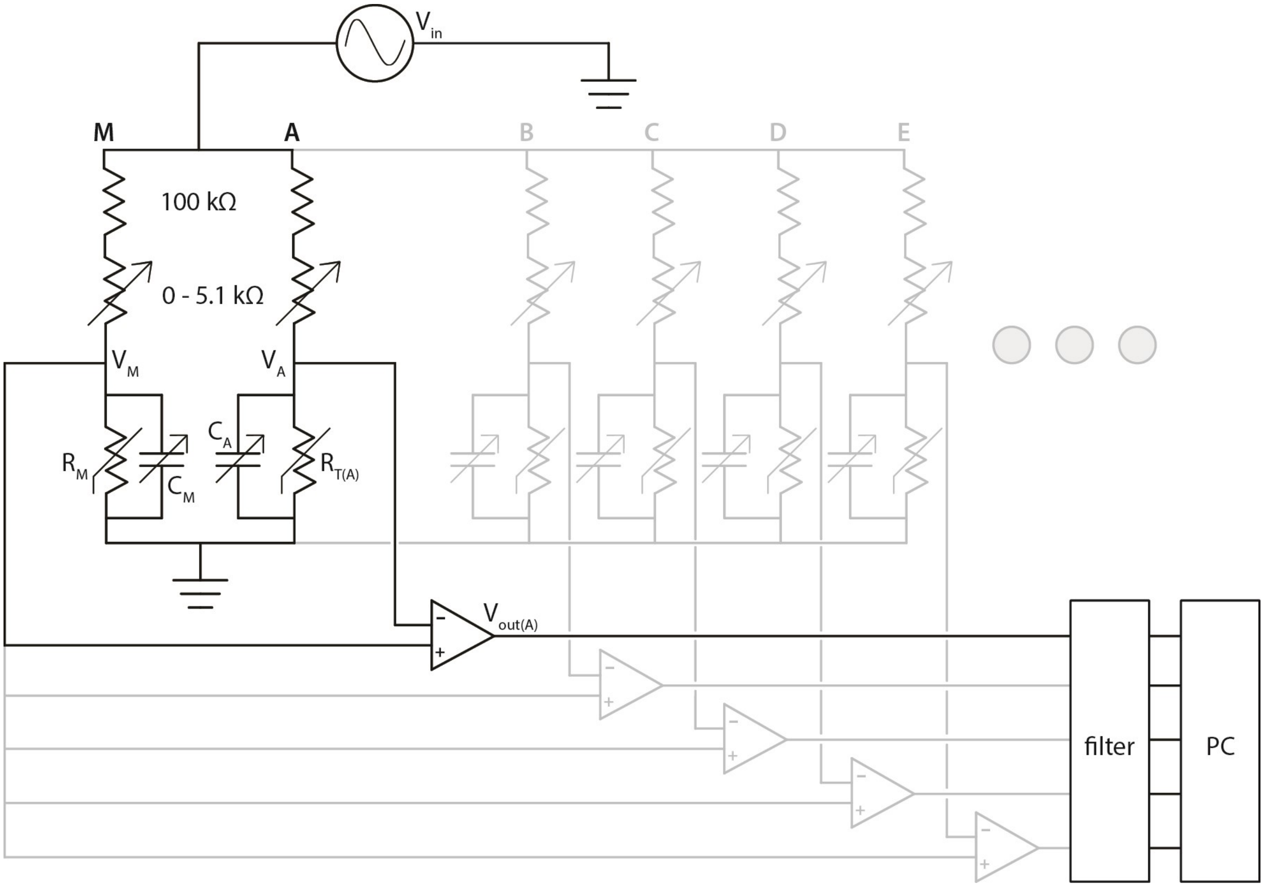

Supplementary Fig. S5 | Circuit diagram for the calorimetry technique. When the resistance of the sensing thermistor $R_{T(A)}$ changes due to a fly's heat production at the center of the tube, an ac voltage difference V_A-V_M develops across the bridge. The ac voltage $V_{out(A)} = 100\times(V_A\text{-}V_M)$ is filtered and deconvolved using an FFT-based algorithm scripted in MATLAB. If, however, the resistance of the sensing thermistor $R_{T(A)}$ changes due to ambient temperature drift, then similar changes in the resistance of the matching thermistor R_M keep the bridge voltage V_A-V_M balanced, thus canceling the detrimental drift signal. See Online Methods and **Supplementary Fig. S6b, c**.

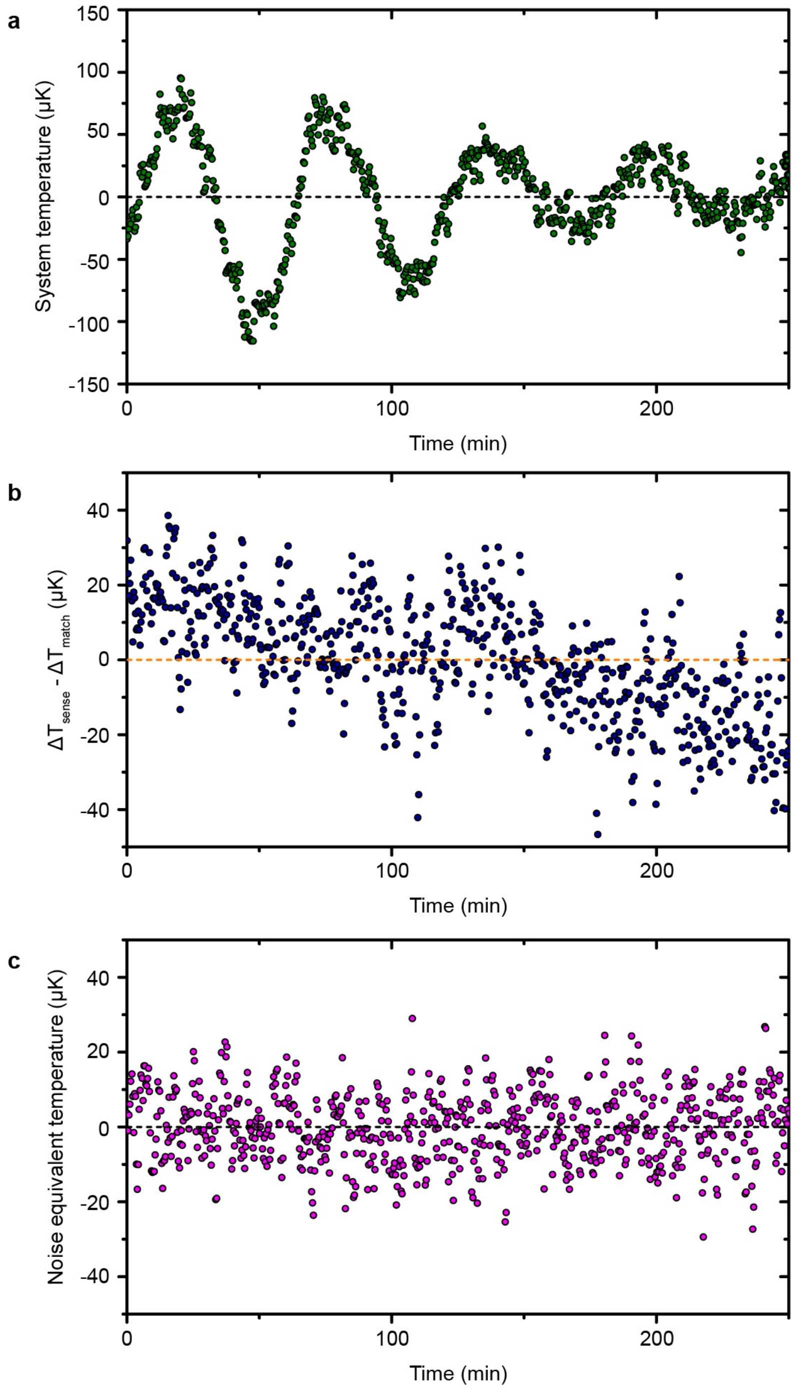

Supplementary Fig. S6 | System and circuit stability characterization. (**a**) Temperature drift at the base of one glass tube over a four-hour timespan. The thermistor used in this measurement is independent of the PID control circuit. The inner shield is seen to be stable to within ±100 μK. (**b**) Drift of the calorimeter signal over a four-hour timespan. For this characterization, the tube was empty. The temperature signal is stable to within ±30 μK, which corresponds to heat flows of ~100 nW assuming a tube conductance of 2 mW/K. (**c**) The noise equivalent temperature ΔT_{NET} of the calorimeter circuit plotted over four hours. The ΔT_{NET} was measured by replacing both sensing and matching thermistors with stable, fixed resistors and logging the bridge voltage difference, which is stable to within ±17 μK, or equivalently 85 nW.

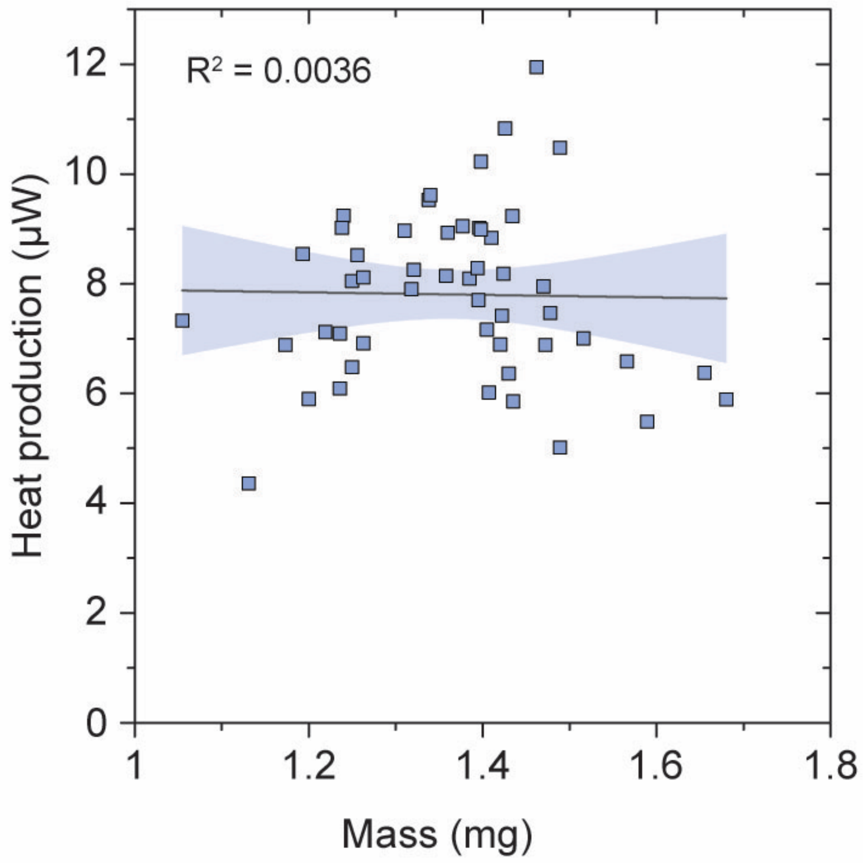

Supplementary Fig. S7 | Heat production was not correlated with body mass. Body mass plotted on the x -axis and heat production plotted on the y-axis for individual *Canton S* 3-day old females. Heat production and mass are not correlated ($R^2 = 0.0036$) for these flies.

Supplementary Table S1: Sugar yeast fly media ingredients			
	Restricted	*Normal*	*High Calorie*
Component	*Amount*	*Amount*	*Amount*
Water (1)	750 ml	750 ml	750 ml
Water (2)	250 ml	250 ml	250 ml
Agar	21 g	21 g	21 g
Sucrose	50 g	100 g	150 g
Yeast	50 g	100 g	150 g
20% Tegosept	15 ml	15 ml	15 ml
Propionic Acid	3 ml	3 ml	3 ml

Supplementary Table S1 | *Drosophila* food media. To prepare the sugar yeast food media, water (1) and agar are combined in a large kettle. The solution is simmered under slow mixing for 40 min. We then combine water (2), yeast, sucrose, dextrose (MP Biomedicals), and cornmeal (SYSCO Corp.) in a separate container and mix well. This mixture is added to the agar solution, and mixing speed is increased while the food is boiled for 15 min. Heat is removed and the food is allowed to cool to 65°C, after which tegosept and propionic acid (Genesee Scientific) are added, and the food is dispensed into 150 ml bottles or 28.5×95 mm vials.
